# Editorial: Case reports in heart valve disease: 2022

**DOI:** 10.3389/fcvm.2023.1260522

**Published:** 2023-08-02

**Authors:** Maximillian A. Rogers, Giuseppe Tarantini, Verena Veulemans

**Affiliations:** ^1^ Department of Medicine, Harvard Medical School, Boston, MA, United States; ^2^ Department of Cardiac, Thoracic and Vascular Sciences and Public Health, University of Padova, Padova, Italy; ^3^ Department of Cardiology, Pulmonology, and Vascular Diseases, University Hospital Düsseldorf, Düsseldorf, Germany

**Keywords:** aortic valve disease, TAVR, infective endocarditis, rheumatic heart diease, mitral valve, aortic stenosis, coronary artery disease

**Editorial on the Research Topic** Case reports in heart valve disease: 2022

Heart valve disease affects tens of millions of people worldwide (1), greatly impacting loss of function, quality of life, and mortality. Each year we learn more about conditions impacting patients with the aim of better identifying, treating, and reducing this significant global health burden. 2022 continued this aim, with 10 interesting case reports being published that enhanced our knowledge of these areas, summarized in Table 1. Heart valve disease can originate from bacterial infections as well as valve functional and degenerative causes. In low- and middle-income countries, rheumatic heart disease that results from damage to heart valves caused by rheumatic fever incidents is the most common form of heart valve disease. Whereas functional and degenerative valvular diseases predominate in high-income countries (1). The case reports in this collection span these conditions, giving a large array of heart valve disease causes, identification, and treatment recommendations relevant to heart valve clinicians and scientists around the world. A high impact was reached by this collection with thousands of article views and downloads in the past year.

**Table 1:** Summary of 2022 case reports in heart valve disease.

Heart valve disease	Article title and reference	Key points and implications
Rheumatic heart disease	Surgical valvular pulmonary reconstruction for a previous unreported rheumatic right-sided valve disease with severe pulmonary regurgitation Zhou et al.	A very rare case of rheumatic right-sided valve disease with severe pulmonary valve contracture and regurgitation was successfully managed with surgical valvular reconstruction
Infective endocarditis	Infective endocarditis cause by Streptococcus sinensis: the first case in mainland China and literature review Zhang et al.	*Streptococcus sinensis* isolated from a young patient with infective endocartisis in mainland China, supporting that may be an emerging pathogen of interest
Native valve endocarditis and valve prostheses endocarditis	Treatment of left-sided valve endocarditis using the transapical AngioVac System and cerebral embolism protection device: a case series Fiocco et al.	Combined use of AngioVac System and cerebral embolism protection system Triguard may be useful for treating left native valve and valve prostheses endocarditis in prohibitive-surgical-risk patients
Aortic stenosis with coronary artery disease	Transcatheter aortic valve replacement in patients undergoing robotic totally endoscopic coronary artery bypass: a case series Srivastava et al.	Demonstration of a hybrid approach treating aortic stenosis with TAVR under conscious sedation prior to robotic off-pump totally endoscopic coronary artery bypass graft surgery as an effective treatment
Aortic valve replacement complication	Paravalvular regurgitation post transcatheter aortic valve replacement: when in doubt choose cardiac magnetic resonance Hadley et al.	Cardiac magnetic resonance provides a more accurate assessment of paravalvular leak severity following TAVR
Aortic valve replacement complication	Severe structural valve deterioration after TAVR with ACURATE Neo: report of two cases Schaeffer et al.	Structural valve deterioration of TAVR prostheses is an uncommon complication that can occur
Aortic valve replacement complication	Emergently alteration of procedural strategy during transcatheter aortic valve replacement to prevent coronary occlusion: a case report Dai et al.	Comprehensive assessment of coronary risk should be made prior to TAVR. A short-stent prosthesis is feasible for patients with high coronary occlusion risk; however, TAVR should be called off when extremely high risk coronary obstruction is identified and no solution can be found
Quadricuspid aortic valve	Surgical repair of a quadricuspid aortic valve with severe regurgitation utilizing “tricuspidization” and annular banding: a case and technique details report Yu et al.	In a rare case a patient with flexible and reservable cusps allowed for aortic root reconstruction using a tricuspidization and annular banding technique
Surgical tricuspid valve repair complication	Transcatheter edge-to-edge repair after prior surgical tricuspid annuloplasty Afzal et al.	Tricuspid transcatheter edge-to-edge repair is an alternative and less invasive option for patients with a failed previous annuloplasty repair for tricuspid regurgitation
Mitral valve regurgitation	Cardiovalve in mitral position additional solution for valve replacement Sherif et al.	Transfemoral mitral valve replacement treated severe mitral regurgitation due to severe restriction of the posterior mitral leaflet

Understanding emerging causes, effective treatments as well as gaps enables the clinical and research communities to best address heart valve disease originating from bacterial infections. Rheumatic heart disease, an autoimmune inflammatory reaction with streptococci has been almost eradicated in several parts of the world. However, it remains the most common cardiovascular disease in children and young people worldwide, impacting vulnerable communities in sub-Saharan Africa, the Middle East, South-East Asia, and Western Pacific. Despite declining rheumatic heart disease burden, the 2015 Global Burden of Disease study estimated 29.7–43.1 million cases and about 300,000 associated deaths (2). Early diagnosis through means like echocardiography when prophylaxis is most likely to be effective in treating patients is a major strategy in managing this disease (3). Beyond early prevention, knowing treatment options likely to benefit patients, particular with less commonly observed complications is of importance. In this collection, Zhou et al. report a successfully managed case of rheumatic right-sided valve disease, a rarely affected tissue that may result in severe rheumatic pulmonary regurgitation, by surgical valvular reconstruction.

Infective endocarditis occurs by infection of the endocardial surfaces of the heart and can be fatal if not treated. The annual incidence is estimated at 3–10/100,000 and has a mortality of up to 30%, with *Staphylococcus aureus* being the most prevalent cause followed by streptococci infection (4). Zhang et al. report a case of infective endocarditis in mainland China associated with streptococcus sinensis, contributing to an increasing number of cases reported with this emerging pathogen. Surgery is used in acute heart failure following large vegetations, but a substantial number of patients have high surgical risk necessitating alternatives that have low or acceptable risk for patients. One alternative is the AngioVac System for mass removal. Also in this collection of case reports, Fiocco et al. present a case series with AngioVac System utilization and the cerebral embolism protective system Triguard. These authors validate this hybrid approach by treating prohibitive-surgical-risk patients in a way that reduces cerebral embolization risk stemming from this mass removal system.

Transcatheter aortic valve replacement (TAVR) is a minimally invasive procedure used frequently in high-income countries to treat aortic stenosis patients who are at risk for death from surgery. TAVR has also been suggested to be noninferior to surgery in low-surgical-risk patients (5). TAVR may also be combined with other procedures, as exemplified by Srivastava et al., who report a case series in which TAVR was safely completed prior to coronary revascularization for patients with coronary artery disease and aortic stenosis. Although highly effective, TAVR is not entirely without risk. Paravalvular leak is a complication that can follow TAVR, and one with which Hadley et al. provide support of using cardiac magnetic resonance to more accurately assess. While uncommon, structural valve deterioration of TAVR prostheses is another concern, as shown in the cases reported by Schaeffer et al. in this collection. Coronary occlusion is another uncommon but fatal complication of TAVR, which Dai et al. highlight the importance of performing a comprehensive coronary risk assessment to avoid. The management of coronary artery disease with severe aortic stenosis is particularly important with extension of transcatheter aortic valve implantation to younger and lower-risk patients, discussed in detail in a consensus statement on this topic (6). Quadricuspid aortic valve is a rare congenital disease, in which most patients are treated with aortic valve replacement. However, valve reconstruction can be an alternative to replacement. In this collection, Yu et al. report a case in which tricuspidization and annular banding technique was applied in a patient with cusps that allowed for corrective reconstruction. In addition to the aortic valve, both surgical and minimally invasive procedures are used to treat patients with other forms of heart valve disease. Surgical tricuspid valve repair is another lifesaving procedure but is also with risk. Tricuspid regurgitation may occur following surgical tricuspid valve repair, and Afzal et al. report a case where tricuspid transcatheter edge-to-edge repair was successfully used in a patient with massive tricuspid regurgitation after surgery. Mitral regurgitation is similarly a risk factor for mortality, and Sherif et al. support minimally invasive transcatheter mitral valve replacement to correct this condition.

Knowing the major and emerging causes of heart valve disease, and how to effectively treat them with minimal risk to patients is paramount to achieving declines in patient mortality. While the reports here represent a single or in some instances a small series of cases, they add to that larger goal by providing the heart valve community with greater knowledge on rare conditions as well as best practices to reduce patient treatment risks.

## References

[1] Coffey S Roberts-Thomson R Brown A Carapetis J Chen M Enriquez-Sarano M Global epidemiology of valvular heart disease. Nat Rev Cardiol. (2021) 18:853–64. 10.1038/s41569-021-00570-z 34172950

[2] Watkins DA Johnson CO Colquhoun SM Karthikeyan G Beaton A Bukhman G Global, regional, and national burden of rheumatic heart disease, 1990–2015. N Engl J Med. (2017) 377:713–22. 10.1056/NEJMoa1603693 28834488

[3] Kumar RK Antunes MJ Beaton A Mirabel M Nkomo VT Okello E Contemporary diagnosis and management of rheumatic heart disease: implications for closing the gap: a scientific statement from the American heart association. Circulation. (2020) 142:e337–57 10.1161/CIR.0000000000000921 33073615

[4] Rajani R Klein JL. Infective endocarditis: a contemporary update. Clin Med (Lond). (2020) 20:31–5 10.7861/clinmed.cme.20.1.1 31941729 PMC6964163

[5] Popma JJ Deeb GM Yakubov SJ Mumtaz M Gada H O’Hair D Transcatheter aortic-valve replacement with a self-expanding valve in low-risk patients. N Engl J Med. (2019) 380:1706–15 10.1056/NEJMoa1816885 30883053

[6] Tarantini G Tang G Fovino LN Blackman D Van Mieghem NM Kim WK Management of coronary artery disease in patients undergoing transcatheter aortic valve implantation. A clinical consensus statement from the European association of percutaneous cardiovascular interventions in collaboration with the ESC working group on cardiovascular surgery. EuroIntervention. (2023) 19:37–52. 10.4244/EIJ-D-22-00958 36811935 PMC10174192

